# Fano Resonances in Metal Gratings with Sub-Wavelength Slits on High Refractive Index Silicon

**DOI:** 10.3390/ma16216982

**Published:** 2023-10-31

**Authors:** Abdelhaq Belkacem, Hammou Oubeniz, Hicham Mangach, Muamer Kadic, Noureddine Cherkaoui Eddeqaqi, Abdenbi Bouzid, Younes Achaoui

**Affiliations:** 1 Laboratory of Optics, Information Processing, Mechanics, Energetics and Electronics, Department of Physics, Moulay Ismail University, Zitoune, Meknes B.P.11201, Morocco; ab.belkacem@edu.umi.ac.ma (A.B.); h.oubeniz@edu.umi.ac.ma (H.O.); echerkaoui@hotmail.com (N.C.E.); a.bouzid@umi.ac.ma (A.B.); y.achaoui@umi.ac.ma (Y.A.); 2 Light, Nanomaterials Nanotechnologies (L2n), CNRS-ERL7004, Université de Technologie de Troyes, 10000 Troyes, France; hicham.mangach@gmail.com; 3 Institut FEMTO-ST, UMR6174, CNRS, Université de Bourgogne Franche-Comté, 25000 Besançon, France

**Keywords:** discrete state, continuum state, Fano resonance, surface plasmon, FEM method, RCWA method

## Abstract

The enhancement of optical waves through perforated plates has received particular attention over the past two decades. This phenomenon can occur due to two distinct and independent mechanisms, namely, nanoscale enhanced optical transmission and micron-scale Fabry–Perot resonance. The aim of the present paper is to shed light on the coupling potential between two neighboring slots filled with two different materials with contrasting physical properties (air and silicon, for example). Using theoretical predictions and numerical simulations, we highlight the role of each constituent material; the low-index material (air) acts as a continuum, while the higher-index material (silicon) exhibits discrete states. This combination gives rise to the so-called Fano resonance, well known since the early 1960s. In particular, it has been demonstrated that optimized geometrical parameters can create sustainable and robust band gaps at will, which provides the scientific community with a further genuine alternative to control optical waves.

## 1. Introduction

Recently, there has been a growing interest in Fano resonances (FR) due to their versatile applications in various fields, such as chemical and biological sensor development, filter technology, and the creation of non-linear electro-optical devices [1,2,3,4,5]. In 1935, Ugo Fano originally discovered this type of resonance while observing asymmetric absorption in noble gases [6]. He afterwards provided a theoretical description of this phenomenon within the framework of the quantum autoionization states of atoms [7]. This universal phenomenon occurs in a wide range of systems, including optics and acoustics [8,9,10,11]. In both cases, this resonance arises when two vibratory modes, namely the continuous and discrete states, interact either constructively or destructively, resulting in an asymmetric line shape as a response [7,12,13,14]. This asymmetrical response is widely demonstrated in the case of extraordinary optical transmission, particularly in thin metallic gratings with sub-wavelength apertures. This is related to the interaction between incident light and the plasmonic surface modes that propagate along the metal/dielectric interface. Comparable results have also been observed in systems involving elastic waves, such as slit gratings or phononic crystals, as a byproduct of the interaction between elastic waves propagating in the structure and localized resonance modes obtained at specific locations within it [15,16,17]. Wood’s anomalies, which induce a dramatic shift in reflected intensity observed for a small variation in wavelength, have drawn the interest of many researchers [18]. Rayleigh explained that these anomalies may result from the appearance or absence of order diffracted by the grating [19]. Nevertheless, this suggestion was proved insufficient to fully explain the anomalies observed by Wood. The latter conducted in-depth studies in 1912 [20] and then in 1935 [21] to quantify the contribution of surface waves [22]. It was not until 1965 that Hessel and Oliner identified the dual origins of Wood’s anomalies as the emergence of diffracted orders and the involvement of the resonance phenomena, particularly through evanescent surface waves [23]. Furthermore, Maystre and Nevière conducted additional investigations into Wood’s anomalies, using calculations involving poles and zeros of the reflection and transmission coefficients [24]. Porto et al. in 1999 focused on examining the transmission properties of arrays of narrow slits, observing an exceptionally high transmission peak, up to 90% in TM polarization [25]. Collin et al. presented a theoretical demonstration indicating that this kind of structure can achieve extremely high-quality factors, ranging from 10 to $10^{5}$ [26]. Ebbesen et al.’s experiment significantly advanced the study of metallic gratings with sub-wavelength apertures, demonstrating extraordinary transmission in a metallic film perforated with fine holes deposited on a quartz substrate [27]. This experiment revealed an exceptional transmission enhancement that surpassed the predictions of Bethe’s theory [28]. Moreover, this exceptional transmission is attributed to the excitation of surface plasmon resonance [29,30,31], which arises from the coupling between an electromagnetic wave and bound electrons located on the surface of a metal [32]. In 2006, Moreno et al. introduced a configuration capable of generating extraordinary transmission in TE polarization, comprising a suspended metal grating with two thin dielectric layers deposited on both sides [33]. In general, both optical and acoustic Fano resonances share similarities in their underlying physical mechanisms while differing in terms of frequency and wave propagation properties. In this study, our focus is on Fano resonances observed within metallic arrays of slits.

In this arrangement, the surface waves that emerge at the grating’s surface are no longer plasmons, but rather guided waves within the dielectric layers. In this study, we aim to engineer a heterogeneous plate with sub-wavelength apertures capable of inducing broad optical transmission reductions characterized by W-shaped Fano resonances. To achieve this, we employ a silver (Ag) metallic plate with double apertures made of air and silicon (Si). Air and silicon are chosen to model continuum and discrete states, respectively, while silver serves as the plasmonic material within the specified frequency range. Subsequently, we comprehensively investigate various parameters that influence the positions of Fano resonances and the diffraction limit. Additionally, we establish a mathematical framework based on rigorous coupled-wave analysis (RCWA) for comparative analysis with numerical results obtained using the finite element method (FEM).

## 2. Analysis and Modeling

In the pursuit of comprehending the alterations in both the transmission amplitude and phase characteristics of the heterogeneous sub-wavelength grating, our investigation encompasses an array of parameters, including wavelength and various geometrical attributes—such as the grating period, slit width, grating thickness, angle of incidence, and the materials employed. Notably, since the studied structure has significantly smaller slit width and the period in comparison to the incident wavelength, our major focus lies exclusively on the investigation of the transmitted zeroth order. To address this, we have developed a homemade code based on the RCWA. This method offers a high degree of flexibility in adjusting polarization, angle of incidence, and slit dimensions compared to the approach developed by M.G. Moharam [34]. Additionally, the RCWA method, implemented in MATLAB by [35], is employed to model different orders of transmitted and reflected waves by a one-dimensional binary grating in either TE or TM polarization. Our approach, on the other hand, involves projecting the solution of the diffraction problem onto a truncated basis of spatial harmonics, which leads to the formulation of a system of equations for each harmonic.

### 2.1. Design and Excitation Source

**Figure 1:**
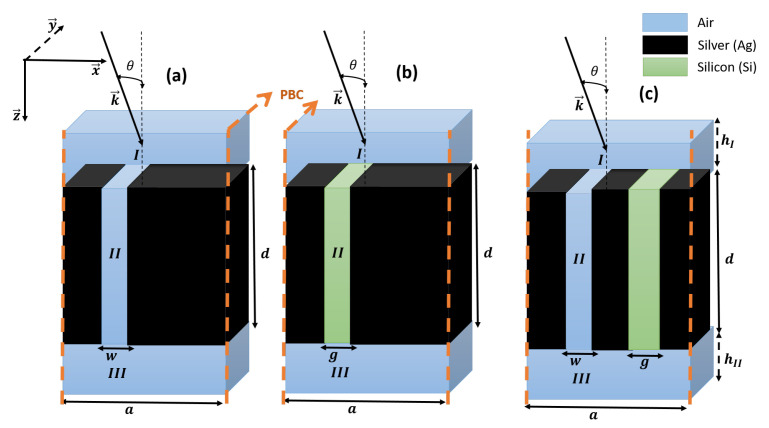
A three-dimensional schematic of the studied unit cells. Panel (**a**) showcases a perforated air slit within a silver (Ag) metallic plate, while panel (**b**) exhibits the deposition of a silicon (Si) slit onto a silver (Ag) metallic plate. Panel (**c**) illustrates the incorporation of two slits (Si and air) within a silver (Ag) metallic plate.

Figure 1 depicts three different geometric patterns explored in this work. Figure 1a shows a silver grating unit cell with an air slit to generate a continuous state, while Figure 1b displays a silver grating unit cell with a silicon slit to establish a discrete state. Figure 1c illustrates a grating unit cell with both air and silicon slits, constituting the final structure under consideration. The latter involves the interaction of continuous and discrete states and plasmons, resulting in a multi-broadband transmission decrease in the form of Fano resonance with a W-line shape.

Furthermore, all the structures have the same periodicity of *a* but feature different slits distinguished by their respective widths: *w* for the air slit and *g* for the silicon slit. The overall geometrical parameters are summarized in Table 1.

These results were obtained through numerical simulations employing FEM and were compared to the RCWA method. In this context, our study pertains to the structure depicted in Figure 1c under a TM-polarized incident plane wave forming an angle θ with (oz)-axis (corresponding to normal incidence). In such a case, the complex amplitude of the magnetic field is given as follows:

(1) $$H_{y,\mathrm{inc}}=H_{y}\left(x,z\right)e^{-j\omega t}=e^{-\left[j\omega t+jk_{0}n_{I}\left(\sin\left(\theta x\right)+\cos\left(\theta z\right)\right)\right]}$$

The incident medium, typically composed of air, is referred to as the superstrate and is identified by the index $n_{I}$, with its permittivity described as $\epsilon_{I}=n_{I}^{2}$. This specific region of space is designated as Region I. The grating region, referred to as Region II, is composed of alternating media with indices $n_{\mathrm{IIm}}$ (permittivity $\epsilon_{\mathrm{IIm}}$ = $n_{\mathrm{IIm}}^{2}$), $\varepsilon_{\mathrm{II},\mathrm{Air}}$ = $n_{\mathrm{II},\mathrm{Air}}^{2}$, and $\epsilon_{\mathrm{II},\mathrm{Si}}$ = $n_{\mathrm{II},\mathrm{Si}}^{2}$. In Region III, situated below the perforated grating, we denote the index of the substrate as $n_{\mathrm{III}}$ (permittivity $\epsilon_{\mathrm{III}}$ = $n_{\mathrm{III}}^{2}$). Furthermore, the expression of the magnetic field amplitude $H_{y}\left(x,z\right)$ differs according to the region considered. In regions I and III, we utilize the Rayleigh decomposition to describe the amplitude of the magnetic fields, $H_{y}\left(x,z\right)$, as described in [36,37].

(2) $$H_{y,I}=H_{y,\mathrm{inc}}+\sum_{n}R^{\left(n\right)}\exp\left[-j(k_{x}^{\left(n\right)}x-k_{z,I}^{\left(n\right)}z)\right]$$

(3) $$H_{y,III}=\sum_{n}T^{\left(n\right)}\exp\left[-j(k_{x}^{\left(n\right)}x+k_{z,II}^{\left(n\right)}\left(z-d\right))\right]$$

where $R^{\left(n\right)}$ and $T^{\left(n\right)}$ represent the complex amplitudes characterizing wave reflections within medium I and wave transmissions within medium III. The periodic structuring along the (OX) axis necessitates the quantization of the tangential component of the wave vector, denoted as $k_{x}^{\left(n\right)}$, for a specified angle of incidence θ. This quantization presents a local manifestation of Snell’s law due to the heterogeneity of the structure, as expressed in Equation (4):

(4) $$k_{x}^{\left(n\right)}=k_{0}n_{I}\sin\left(\theta \right)-n\frac{2\pi }{a}$$

The conservation of momentum is described by:

(5) $$k_{x}^{{\left(n\right)}^{2}}+k_{Zi}^{{\left(n\right)}^{2}}=n_{i}^{2}k_{0}^{2}$$

From Equations (4) and (5) we can derive:

(6) $$k_{Zi}^{\left(n\right)}=\left\{\begin{array}{cc} \sqrt{n_{i}^{2}k_{0}^{2}-{\left(k_{x}^{\left(n\right)}\right)}^{2}}, & \mathrm{if}\;n_{i}^{2}k_{0}^{2}\geq {\left(k_{x}^{\left(n\right)}\right)}^{2},\\ j\sqrt{{\left(k_{x}^{\left(n\right)}\right)}^{2}-n_{i}^{2}k_{0}^{2}}, & \mathrm{if}\;n_{i}^{2}k_{0}^{2}<{\left(k_{x}^{\left(n\right)}\right)}^{2}. \end{array}\right.$$

where $n_{i}$, $k_{0}=\frac{2\pi }{\lambda }$, and $k_{x}^{\left(n\right)}$ represent the refractive index of the medium (i = I, II or III), the magnitude of the wave vector in vacuum, and the tangential wave vector component, respectively, with *n* indicating the order diffracted by the grating. In Equation (2) and Equation (3), the terms $R^{\left(n\right)}$ and $T^{\left(n\right)}$ denote the complex amplitudes of the reflected and transmitted diffracted orders. Our specific focus is on determining the complex values of $T^{\left(0\right)}$. This term represents the amplitude transmitted in order zero, which is the only diffraction order considered when examining structures with a period smaller than the incident wavelength. Moreover, the corresponding expressions for the electric field in regions I and III are directly obtained from the Maxwell–Ampère equation.

### 2.2. The Rigorous Coupled Wave Analysis (RCWA) Method

**Figure 2:**
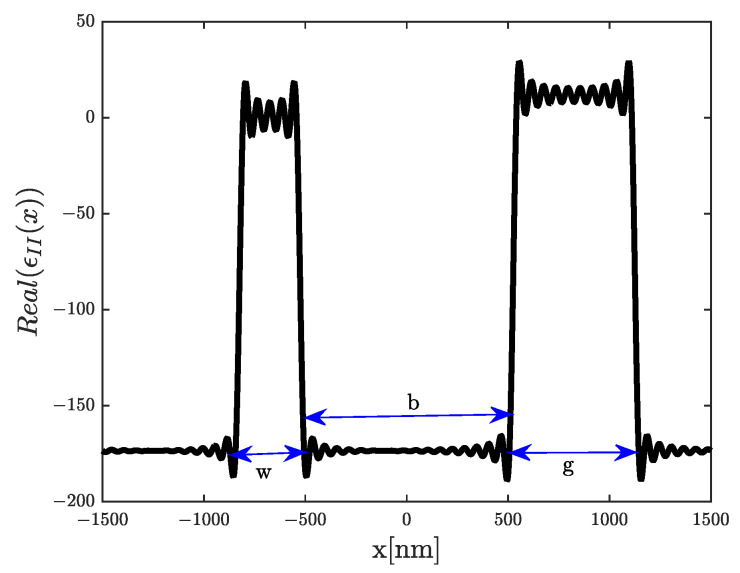
Profile of the real part of the dielectric permittivity obtained by Fourier series decomposition (N=150) of the grating considered in Figure 1c, with the set of geometrical parameters as follows: a=3μm, d=a, $w=\frac{a}{10}$, and $g=\frac{a}{5}$.

In this part, we will employ the RCWA method to analyze the structure illustrated in Figure 1c. Figure 2 depicts the real part of the dielectric permittivity profile for this double-slit periodic grating.

Historically, the RCWA method was developed by Moharam and Gaylord in the early 1980s [38,39], representing a significant advancement in the field of diffractive optics. Initially, it was used to analyze planar structures featuring sinusoidal modulation, whether composed of dielectric or metallic materials [40,41]. Over time, this method was extended to encompass structures characterized by surface reliefs [38] and intricate three-dimensional configurations [39]. Moreover, the RCWA method relies on the Fourier series decomposition of permittivity, a mathematical concept initially introduced by Burckhardt [42], Kaspar [43], and Knop [44] (referred to as the BKK method). Moharam and Gaylord further enriched this approach by incorporating a model that describes the electromagnetic fields within the modulated region as coupled waves, propagating orthogonally to the plane of the grating and corresponding to the diffraction orders emanating from outside the grating structure. A notable feature of the RCWA method is its departure from the conventional second derivative approximation of the electromagnetic field [45]. The permittivity of the structure with respect to the *z*-axis in the three regions, I, II, and III, is given by the following equation, Equation (7):

(7) $$\epsilon \left(x,z\right)=\left\{\begin{array}{cc} \epsilon_{I} & \mathrm{if}\;z>d\\ \epsilon_{II}\left(x\right) & \mathrm{if}\;0\leq z\leq d\\ \epsilon_{III} & \mathrm{if}\;z<0 \end{array}\right.$$

Consequently, in Region II, we perform a Fourier decomposition of the dielectric function $\epsilon_{II}\left(x\right)$ as follows:

(8) $$\epsilon_{II}\left(x\right)=\epsilon_{\mathrm{eff}}+\sum_{n=-N}^{n=N}\epsilon^{\left(n\right)}\exp\left(j2\pi \frac{n}{a}\right)$$

The integer *N* is the number of harmonics retained in the Fourier decomposition. Where,

$$\epsilon_{\mathrm{eff}}=\epsilon_{\mathrm{II},\mathrm{air}}f_{1}+\epsilon_{\mathrm{II},\mathrm{Si}}f_{2}+\epsilon_{\mathrm{II},m}f_{3}$$

and,

$$\epsilon^{\left(n\right)}=-\frac{1}{n\pi }\left[\left[\left(\epsilon_{\mathrm{II},m}-\epsilon_{\mathrm{II},\mathrm{air}}\right)\sin\left(n\pi f_{1}\right)e^{jn\pi (f_{1}+\frac{f_{3}}{2})}\right]+\left[\left(\epsilon_{\mathrm{II},m}-\epsilon_{\mathrm{II},\mathrm{Si}}\right)\sin\left(n\pi f_{2}\right)e^{jn\pi (f_{2}+\frac{f_{3}}{2})}\right]\right]$$

In this decomposition, $f_{i}$ represents the filling factor, which can be expressed as follows:

$$f_{i}=\left\{\begin{array}{cc} \frac{w}{a} & \;\mathrm{for}\;\mathrm{Air}\;\mathrm{slits}\;i=1\\ \frac{g}{a} & \;\mathrm{for}\;\mathrm{Si}\;\mathrm{slits}\;i=2\\ 1-f_{1}-f_{2} & \mathrm{for}\;\mathrm{the}\;\mathrm{remainder}\;i=3 \end{array}\right.$$

$\epsilon^{\left(n\right)}$: the *n*-th harmonic in the decomposition of $x\to \epsilon_{II}\left(x\right)$. Within the array, we also perform a generalized Fourier series expansion of the electric (E) and magnetic (H) fields as a function of spatial harmonics.

(9) $$H_{y}=\sum_{p}h_{y}^{\left(p\right)}\left(z\right)\exp\left(jk_{x}^{\left(p\right)}x\right)$$

(10) $$E_{x}=j\sqrt{\frac{\mu_{0}}{\epsilon_{0}}}\sum_{p}e_{x}^{\left(p\right)}\left(z\right)\exp\left(jk_{x}^{\left(p\right)}x\right)$$

$h_{y}^{\left(p\right)}\left(z\right)$, and $e_{x}^{\left(p\right)}\left(z\right)$ represent the normalized amplitudes of the *p*-th spatial harmonics of the magnetic and electric fields, respectively. By incorporating these Fourier developments into Maxwell’s equations, we obtain a system of coupled equations, which can be expressed in a matrix representation as depicted below:

(11) $$\left(\frac{\partial^{2}h_{y}^{\left(p\right)}}{\partial {\left(k_{0}z\right)}^{2}}\right)=\left[EB\right]\left[h_{y}^{\left(p\right)}\right]$$

where, $B=K_{x}E^{-1}K_{x}-I$

*E*: denotes the matrix formed by the spatial harmonics of the effective grating permittivity, whose element (n,p) is equal to $\epsilon^{\left(n-p\right)}$; $K_{x}$: is a diagonal matrix, whose element (n,n) is $\frac{k_{x}^{\left(n\right)}}{k_{0}}$; *I*: is an identity matrix.

By solving the coupled equation and applying the continuity of the tangential components of the magnetic field $H_{y}$ and electric field $E_{x}$ at the two interfaces (I/II) and (II/III), we obtain the expressions of the fields E and H within the grating, enabling the determination of the complex amplitudes $R^{\left(p\right)}$ and $T^{\left(p\right)}$ as well as the associated efficiencies for each order.

- The *p*-order diffraction efficiency in reflection Equation (12) and transmission Equation (13):

(12) $${DE}_{r}^{\left(p\right)}={\left|R^{\left(p\right)}\right|}^{2}\mathrm{Real}\left(\frac{k_{z,I}^{\left(p\right)}}{k_{0}n_{I}\cos\theta }\right)$$

(13) $${\mathrm{DE}}_{t}^{\left(p\right)}={\left|T^{\left(p\right)}\right|}^{2}\mathrm{Real}\left(\frac{k_{z,III}^{\left(p\right)}}{k_{0}n_{I}\cos\theta }\frac{n_{I}^{2}}{n_{III}^{2}}\right)$$

- The transmission spectrum in decibels, denoted as T(dB), is given by Equation (14):

(14) $$T\left(\mathrm{dB}\right)=20\log_{10}\left(T^{\left(p\right)}\right)$$

## 3. Results and Discussion

### 3.1. Design and Characterization of the Continuum State

We compared the results obtained by the (RCWA) method with those of numerical simulations using the finite element method (FEM). The (FEM) method cuts the region of interest into a mesh of small elements (we chose a triangular mesh with a $\frac{a}{10}$ pitch in Regions I and III, while in Region II, we chose an Extremely Fine mesh with a $\frac{w}{10}$ pitch in order to obtain results that converge towards the global solution). Note that the method (FEM) solves Maxwell’s equations by discretizing space into finite elements and using basis functions to approximate electromagnetic fields. The equations are solved locally in each element, then assembled to obtain the global solution. We first generate a plane wave with a wave vector $\vec{k}$ polarized in the transverse magnetic (TM) mode along the structure’s axis (OZ). Transmission is evaluated by comparing the signal passing through the structure with the source signal. Due to the periodic nature of the system, our model is confined to a single unit cell, and Floquet periodic boundary conditions (PBC) are applied along the (OX) axis, as illustrated in Figure 1a. Perfectly matched layers (PML) are also used to reduce reflections on the system boundaries, which simulate an artificial infinite domain. The physical properties of the materials used for simulation are those described in [46] for silver, and for silicon they are extracted from [47].

We start our investigation by examining a one-dimensional (1D) slit within a silver plate. Therefore, the cavity resonances are observed in the transmittance spectrum. We provide analytical formulations to describe the position of these resonances. Furthermore, employing the same theoretical framework, we elucidate the physical mechanisms responsible for the emergence of novel transmission resonances in structures composed of periodic slit arrays. The Fabry–Perot resonators yield transmission enhancements at precisely defined frequencies, where incident wave energy is fully transmitted through the slits. This phenomenon becomes particularly pronounced under rigid conditions, such as when dealing with silver, which exhibits near-perfect metal behavior within the examined frequency range, especially at the edges of the periodic unit.

**Figure 3:**
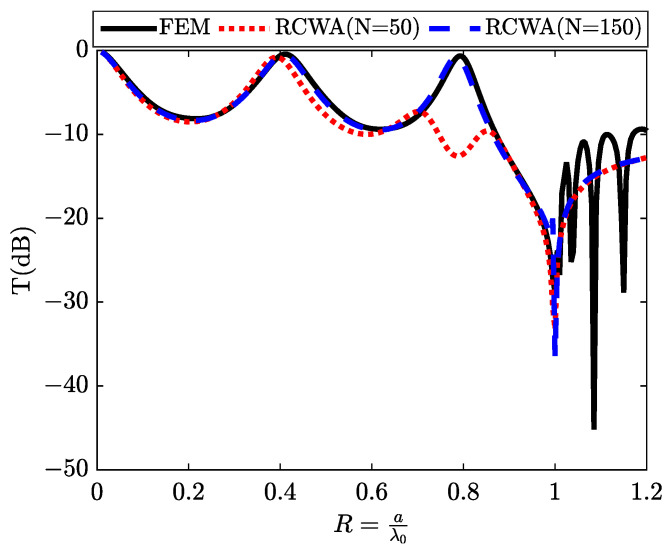
The transmission spectrum T(dB) obtained through an array of air slits, describing the continuum state.

The spectra in Figure 3 reveal two resonances within the transmission spectrum, specifically when the thickness equals the grating period (d = a). Collin et al. carry out an analysis of the impact of the angle of incidence on the spectral position of these peaks [26]. They observe that the peak corresponding to short wavelengths strongly responds to the angle of incidence and follows the surface plasmon dispersion relation calculated for a flat interface. In contrast, the peak associated with longer wavelengths remains nearly invariant, behaving similarly to a cavity resonance. Thus, they propose the existence of two distinct resonant mechanisms: plasmonic resonances along horizontal interfaces and Fabry–Perot resonances within slots (acting as planar wave guides), whose fundamental mode has no cut-off frequency. For an elementary cell with a cross-section of (a×a), two resonances can be obviously identified, corresponding to the guided modes λ and $\frac{\lambda }{2}$, induced by the slit in the metal plate. These resonances manifest prior to the first Rayleigh anomaly, precisely positioned at the reduced frequency $R=\frac{a}{\lambda_{0}}=1$.

The mechanism behind extraordinary transmission through a sub-wavelength metal grating operates as follows: The incident wave interacts with the slit-guided mode, combining partly directly and partly via horizontal surface plasmons on the input face. At the exit, the slit-guided mode couples with a propagating wave, also combining direct interaction and interaction via horizontal surface plasmons on the exit face. The coupling between the plasmons of the input and output faces through the slit is more efficient when the refractive indices of the two media are identical. This is why higher transmissions are obtained with symmetrical structures.

### 3.2. Characterization of Rayleigh Anomalies at Different Angle of Incidence

We will extend our analysis of resonance phenomena by characterizing them in terms of angle of incidence. This involves varying the angle of incidence from 0 to 45 degrees and measuring the transmission spectrum in decibels (dB) for each angle. Transmission minima occur near the appearance or disappearance of a diffracted order. If we denote $k_{x}^{\left(n\right)}$ as the component parallel to the surface of the wave vector diffracted according to order *n* (the refractive index of medium III is given by ($\epsilon_{III}=n_{III}^{2}$)), we have the following relation: $k_{ZIII}^{\left(n\right)}=\sqrt{n_{III}^{2}k_{0}^{2}-{\left(k_{x}^{\left(n\right)}\right)}^{2}}$.

In this equation, we observe that an order appears or disappears when $n_{III}^{2}k_{0}^{2}={\left(k_{x}^{\left(n\right)}\right)}^{2}$. Using the lattice formula provided by Equation (4), we can determine the position of the minimum transmission points: $R=\frac{a}{\lambda_{0}}=\frac{n}{n_{III}+n_{I}\sin\theta }$.

In the case where $n_{III}=n_{I}$, Table 2 provides the positions of the transmission minima for incidence angles of 0°, 15°, 30°, and 45°.

It is noteworthy that all discussions within this section are concentrated exclusively on the resonance phenomena pertaining to the initial Rayleigh anomaly. Analogously to the behavior observed with respect to the transmission minimum, it becomes evident that the position of the maximum transmission peak experiences a redshift toward longer wavelengths with the increment of the incidence angle.

**Figure 4:**
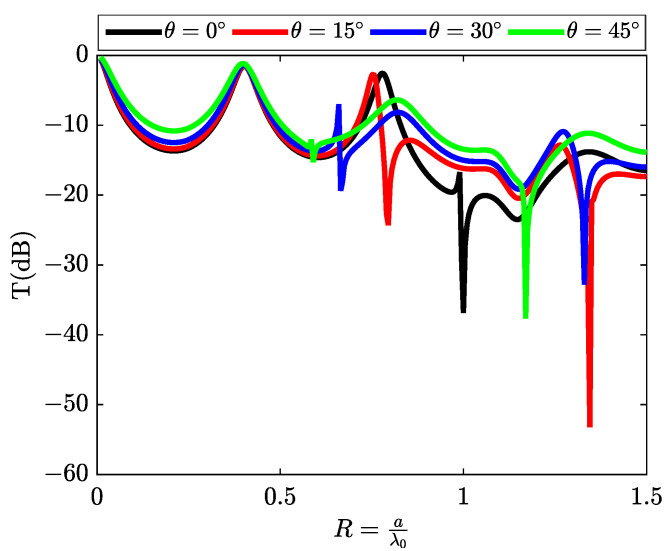
The angular variation in the position of the Rayleigh anomaly for angles of $0^{\circ }$, $15^{\circ }$, $30^{\circ }$, and $45^{\circ }$, respectively.

The curves depicted in Figure 4 elucidate the angular dependency of resonance phenomena within metallic slit gratings. The shifts in peak positions conform to the grating equation, Equation (4), and correspond to the emergence or disappearance of the first-order diffracted wave. It is noteworthy, however, that the resonance peaks manifest an asymmetric line shape when the dimensionless parameter *R* assumes values ranging from 0.6 to 1. Moreover, these plots represent the transmission (dB) spectra for two specific scenarios: normal incidence and incidence angles of 15°, 30°, and 45°. It is discernible that the resonance characteristics, comprising their positions, peak amplitudes, and sharpness, undergo variations with altering angles of incidence. These empirical findings are further substantiated by rigorous calculations conducted via the (RCWA) method.

### 3.3. Discrete State Design

**Figure 5:**
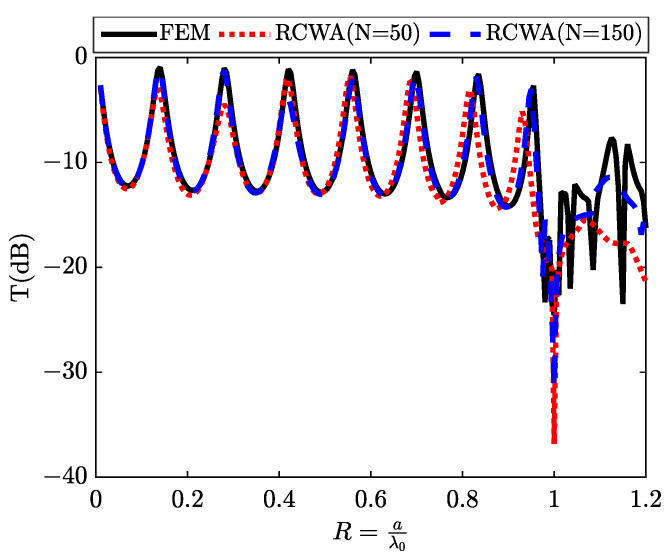
The transmission spectrum T(dB) obtained through an array of silicon slits, describing the discrete states.

We will now endeavor to induce discrete states by replacing the air slit with silicon, which possesses a refractive index over three times greater than that of air. The simulation depicts a metallic grating, as shown in Figure 1b, featuring sub-wavelength silicon slits. This configuration allows us to visualize what we term as discrete states. Figure 5 illustrates the transmission results following the interaction with a metallic array of silicon slits under TM polarization at normal incidence. We exclusively assess the zeroth transmitted order, which, for wavelengths exceeding the grating period, represents the only propagating order. A transmission minimum is observed for a wavelength closely matching the grating period ($a=\lambda \Rightarrow R=\frac{a}{\lambda }=1$), corresponding to the threshold of a new propagated diffracted order’s emergence. At longer wavelengths, we observe prominent transmission peaks, approaching nearly 90%. These remarkable transmission outcomes affirm the system’s exceptional quality. The dotted curves within the same figure display the results of calculations conducted using the (RCWA) method for two cases (N = 50) and (N = 150), consistent with the results obtained through numerical simulation (solid line) based on the (FEM) method.

### 3.4. Coupling between Continuum and Discrete States

**Figure 6:**
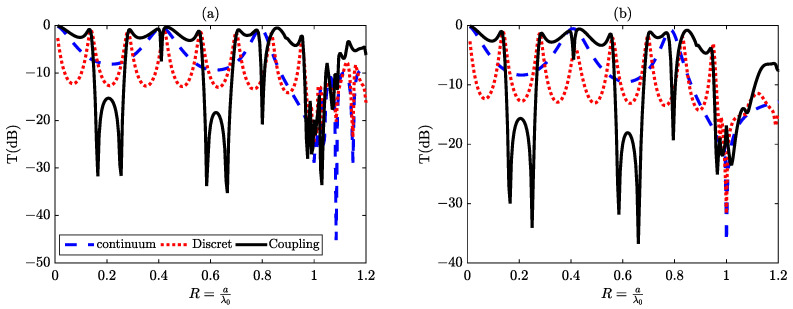
The transmission spectrum in (dB) represents the coupling between the continuum state and the discrete states, as obtained through two methods: (**a**) using the (FEM) method and (**b**) using the (RCWA) method with N = 150.

In this section, the resonances we have just observed have the particularity of showing a transmission minimum close to a transmission maximum. This type of resonance was described by Fano in reference [7] to explain the spectrum of the helium atom resulting from interference between its different electronic states during the auto-ionization phenomenon. In optical systems such as those we are studying, Fano resonances appear to arise from interference between two transmission paths of the incident wave through the structure. In the scenario where a unit cell comprises an air gap of width w, separated by a distance b from another silicon gap of width g (refer to Figure 1c), a coupling effect emerges between the continuum state generated by the air gap and the discrete states produced by the silicon gap. This interaction results in the manifestation of two asymmetric peaks within the transmission spectrum, indicative of a Fano resonance phenomenon. These resonances manifest within the reduced frequency ranges denoted as $R_{1}\;\mathrm{and}\;R_{2}$, as well as between $R_{4}\;\mathrm{and}\;R_{5}$ as illustrated in Figure 6a,b.

In fact, by utilizing the relationship $2n_{Si}d=p\lambda_{0}$ (where $n_{Si}$ represents the refractive index of silicon), it becomes possible to predict the precise positions of resonances resulting from the silicon slot drilled into the unit cell of the metal lattice with a thickness d. Consequently, the resonance locations can be determined as $R_{p}=\frac{d}{\lambda_{0}}=\frac{p}{2n_{\mathrm{Si}}}$ (where *p* denotes the interference order and $n_{Si}=3.44$), as illustrated in Figure 5 and Table 3.

The most remarkable feature of this type of resonance is its higher quality factor compared to the peak resonances of discrete states, commonly known as Lorentzian resonances. Between these two Fano resonances, a wide band of attenuation occurs around the reduced frequencies corresponding to $R_{1}\;\mathrm{and}\;R_{2}$. This attenuation exceeds the limits of conventional attenuation typically observed with perforated plates in air.

**Figure 7:**
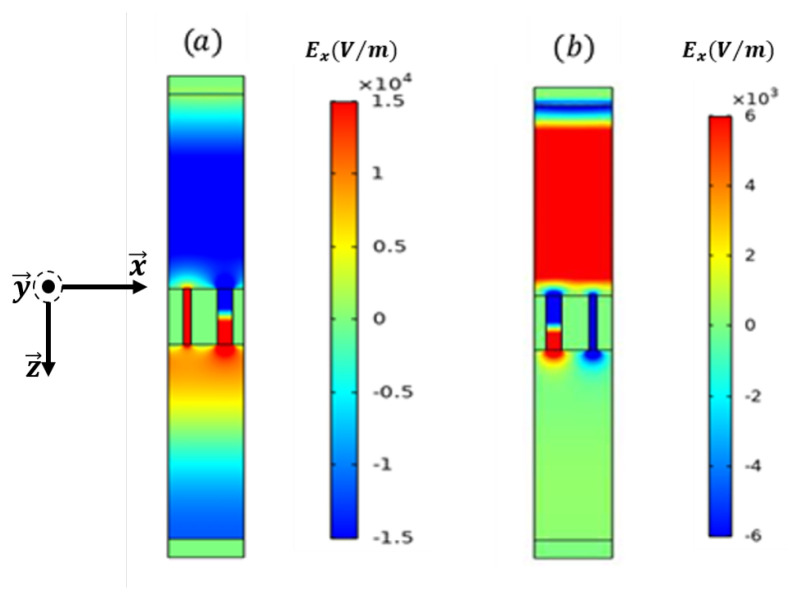
The electric field distribution along the period axis (OX). (**a**) At the resonance and (**b**) anti-resonance modes as manifested in the transmission spectrum. The (OY) axis presents the wave propagation direction under normal incidence.

Figure 7 illustrates the distribution of the electric field component along the (OX) axis for the first resonance and anti-resonance modes of transmission. The resonant mode associated with $R_{1}$ is depicted in Figure 7a. In this case, a pumping motion of the aperture induces high transmission on the opposite side of the structure. At the frequency corresponding to the anti-resonant mode, which occurs just after the first resonant mode, almost total reflection is observed in Figure 7b. Electrical energy remains trapped in the two slots and in the metal, but the wave undergoes a phase shift of 180°, inducing total reflection.

### 3.5. The Effect of the Filling Factor $f_{2}=\frac{g}{a}$ of Silicon (Si) Slits on Resonances

**Figure 8:**
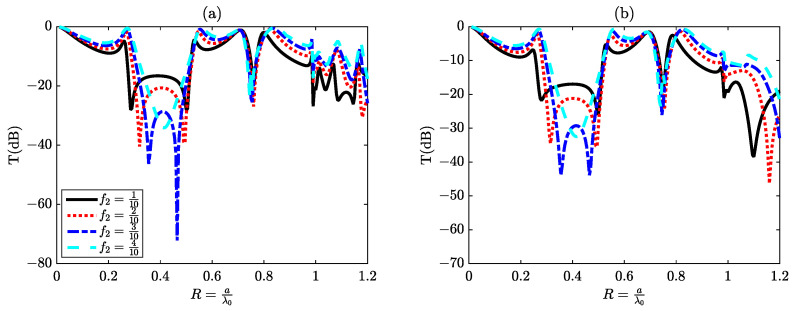
The transmission spectrum as a function of $f_{2}$ ($f_{2}=\frac{g}{a}$, the filling ratio of (Si) slits), obtained by (**a**) the (FEM) Method and (**b**) the (RCWA) method.

In this section, we have chosen an elementary cell characterized by a period a equal to twice its thickness d (a = 2d) as a means to simplify our analytical approach. Within this configuration, we have identified two distinct propagating modes, denoted as $R_{1}$ and $R_{2}$, which manifest before reaching the initial Rayleigh anomaly. The impact of modifying the thickness d on resonance frequencies is a well-documented phenomenon. By diminishing the value of d, we can effectively shift these resonance peaks towards lower frequencies. This adjustment accounts for the disappearance of the resonance peak associated with *p* = 2 in the continuum state, as depicted in Figure 3, along with the attenuation of other resonance peaks corresponding to p>3 in the discrete state, as portrayed in Figure 5. To manipulate the anti-resonance frequencies, we primarily modify the width parameters g or w, which are analogous to ($f_{1}$ or $f_{2}$), of one of the slits. For example, an increase in the slit width g results in a convergence of the two anti-resonance frequencies. The effect of parameter $f_{2}$ is investigated through a systematic examination of transmission spectra denoted as T(dB). These spectra are generated via numerical simulations employing both the (FEM) method and the (RCWA) method. The study incorporates a substantial value for a parameter denoted as N, specifically setting N=150. This choice of N is made in order to assess the convergence of the RCWA method towards the numerical outcomes produced by the FEM method. Notably, as the value of N (representing the number of harmonics) is systematically increased, the results exhibit a strong convergence with those acquired from numerical modeling based on the FEM method, as illustrated in Figure 8.

### 3.6. Scaling Factor

It is important to emphasize that the outcomes mentioned above can be customized for different wavelengths. Achieving consistent transmission results at various wavelengths is theoretically straightforward—simply apply a scaling factor equal to the ratio between the desired wavelength and initial wavelength used in the calculations. By scaling all geometrical parameters of the elementary cell uniformly, one can achieve identical transmission characteristics at different wavelengths. We are currently investigating how the structure’s transmission behavior changes as we scale its period (a) of the unit cell. Notably, all geometrical parameters listed in Table 1 exhibit linear behaviour with the unit cell’s period (a). Therefore, any changes in a directly affect the dimensions of the entire structure. This study focuses on the concept of scaling the unit cell, aiming to elucidate the consequences of scaling—whether through reducing or increasing the unit cell size—by applying a scaling factor to the structure’s optical response. Our primary goal is to determine the minimum scaling factor required to replicate the optical response observed in the earlier sections of this investigation.

**Figure 9:**
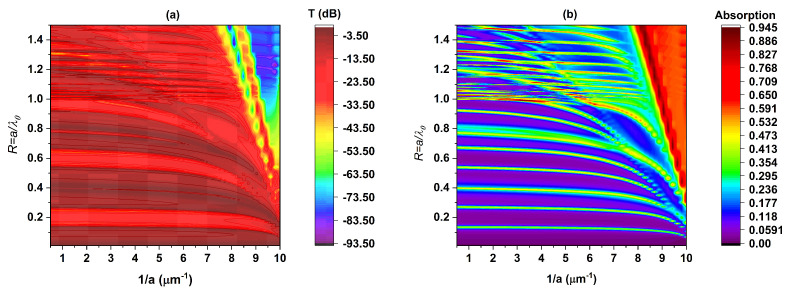
A contour (**a**): of transmission T(dB) and (**b**): of absorption as functions of reduced frequency R and the inverse of the period ($\frac{1}{a}$).

In pursuit of our objective, we deliberately chose to investigate a period variation spanning from 0.1 to 2μm, encompassing a total of 50 discrete values. The results are graphically depicted in Figure 9, which portrays the contour plots of transmission (measured in decibels and absorption against two critical parameters: the reduced frequency denoted as R and the reciprocal of the period denoted as $\frac{1}{a}$. This specific parameter range was carefully selected to yield graphical representations resembling dispersion diagrams akin to those found in photonic structures. From the examination of Figure 9, we are able to draw the following conclusions:

- If $\frac{1}{a}\in \left[0.5,4\right]\Rightarrow a\in \left[0.25,2\right]\;\mu m$:

The attenuation created between the frequencies corresponding to $R_{1}\;\mathrm{and}\;R_{2}$ (centered around R=0.2) and that created between $R_{4}\;\mathrm{and}\;R_{5}$ (centered around R=0.6) retains almost the same values of T(dB) and R, indicating that the structure maintains a coherent response. This allows us to predict the position of resonance peaks for any value of period **a** within this range.

- If $\frac{1}{a}\in \left[4,8\right]\Rightarrow a\in \left[0.125,0.25\right]\;\mu m$:

The attenuation between the frequencies corresponding to $R_{1}\;\mathrm{and}\;R_{2}$ (centered around R=0.2) varies at a slight angle compared to that between $R_{4}\;\mathrm{and}\;R_{5}$ (centered around R=0.6). This indicates that the peaks of the latter are influenced by significant absorption, primarily due to the metal in this frequency range. The impact of absorption on these peaks results in decreased transmittance and a shift toward lower frequencies, eventually leading to the disappearance of the peaks corresponding to $R_{4}\;\mathrm{and}\;R_{5}$.

**Figure 10:**
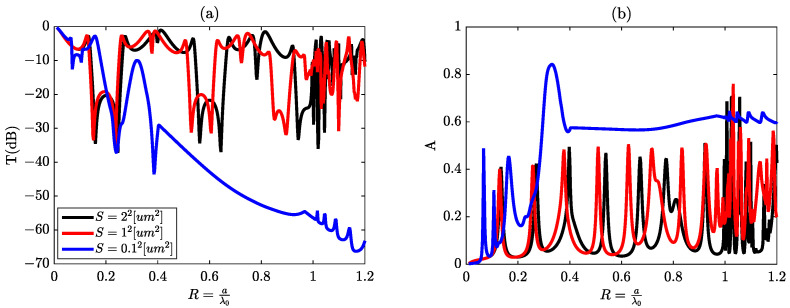
(**a**,**b**) Transmission and absorption spectra with respect to the reduced parameter R, respectively.

- If $\frac{1}{a}\in \left[8,10\right]\Rightarrow a\in \left[0.1,0.125\right]\;\mu m$:

It is notable that only Fano resonances corresponding to $R_{1}\;\mathrm{and}\;R_{2}$ can be generated, albeit with a marginal reduction in attenuation and a simultaneous shift toward lower frequencies. In this context, the behavior of the metallic material exerts a profound influence on the emergence of all coupled states and, consequently, on the generation of all conceivable Fano resonances. This effect of the metallic material is effectively demonstrated in Figure 10a,b: The solid black curve corresponds to a=0.1μm. The blue line corresponds to a=1μm. The continuous green curve corresponds to a=2μm. Upon examining these three curves, the initial observation is that, for a=1μm and a=2μm, the two curves almost coincide within the interval [0,0.3], displaying only a minor shift of approximately δR=0.05 within the interval [0.3,0.7].

Additionally, when considering the curve associated with a dimension of a=0.1μm (section $s=0.1\;\mu m^{2}$), we observe a pronounced shift in resonance peaks towards lower frequencies compared to those occurring for values of *a* greater than or equal to 1,μm. This shift coincides with the disappearance of peaks corresponding to $R_{4}\;\mathrm{and}\;R_{5}$. This intriguing phenomenon emerges when the structural dimensions fall below the threshold of 1,μm. In such cases, the wavelengths associated with the imaginary component of the metal’s refractive index become smaller than those corresponding to the real component. Consequently, the metal undergoes a transition to a transparent state, significantly enhancing the absorption spectrum. This enhancement, in turn, leads to a noticeable shift of the resonance peaks towards lower frequencies.

### 3.7. The Effect of the Substrate on the Response of the Structure

To ensure the feasibility of this structure, the use of a substrate becomes necessary. To streamline the manufacturing process, it is imperative that the substrate shares the same material properties as the silicon slit. As a result, we have opted for a square structure with a periodicity of a. This ensures that the diffraction limit remains outside the two resonance peaks associated with discrete states. The diffraction limit is directly influenced by the periodicity of the lattice. Notably, for a surrounding medium of air and under normal incidence, it occurs at $R=\frac{a}{\lambda_{0}}=1$. However, in the case of a silicon surrounding medium with a refractive index of $n_{Si}=3.44$, this limit shifts to $R=\frac{a}{\left(n_{Si}\lambda_{0}\right)}=\frac{1}{n_{Si}}=0.29$. Based on these two observations, the effect of the silicon substrate on the response of the studied structure is to bring the diffraction limit closer to that of free space by a factor of $\frac{1}{n_{Si}}$.

**Figure 11:**
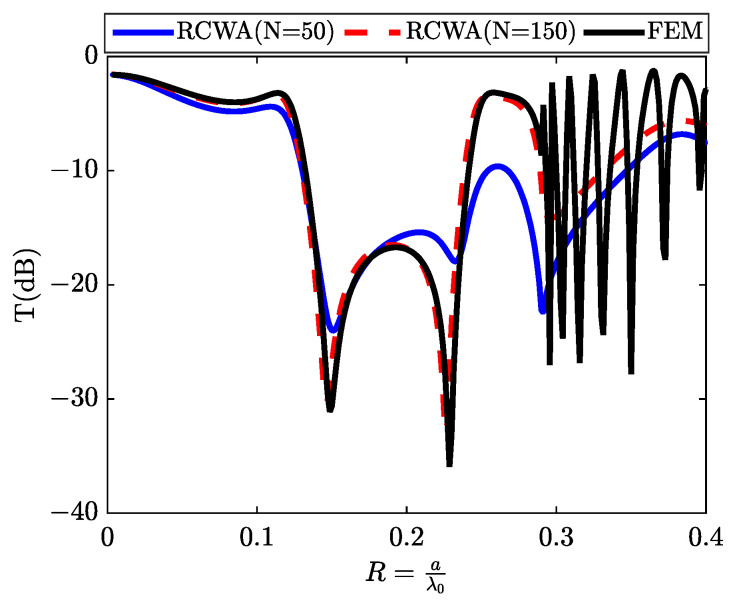
Transmission for the structure shown in Figure 1c, with a silicon (Si) substrate. A comparison between the (FEM) method (solid black line) and the (RCWA) method (dotted blue line N = 50; dotted red line N = 150), respectively.

**Figure 12:**
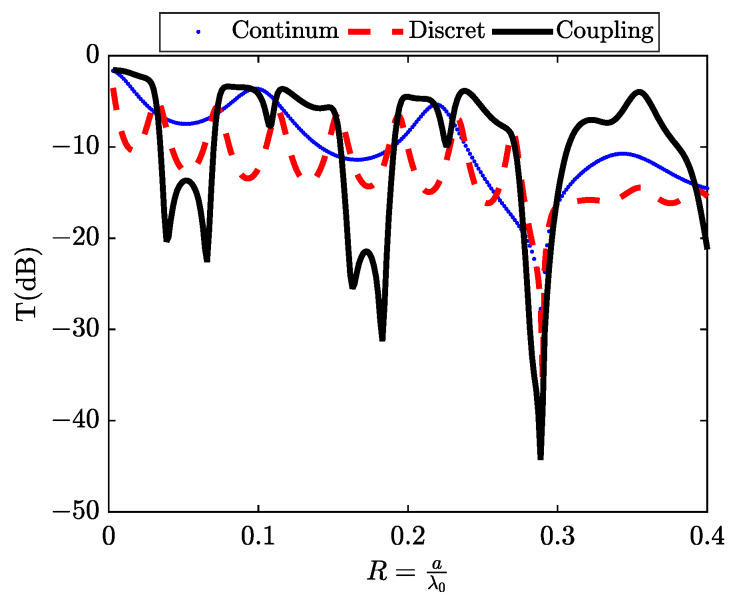
The mechanism of Fano resonance with a W-shape in transmission curve T(dB) is calculated using the (RCWA) method with a number of harmonics of (N = 150). This calculation is performed for a metallic slit grating sub-wavelength structure with the following parameters: period a=3μm, thickness $d^{{}^{\prime }}=n_{\mathrm{Si}}\cdot d=10.32\;\mu m$, air gap $w=\frac{a}{10}$, width of Si Slit $g=\frac{a}{5}$, and a refractive index $n_{III}=n_{Si}=3.44$.

From Figure 11, it is evident that the resonance and anti-resonance peaks occur at the same reduced frequency R as the structure under study, which has dimension a as its period and thickness $d=\frac{a}{2}$, associated with the surrounding free-space medium. This implies that the first two diffraction modes, associated with $R_{1}$ and $R_{2}$, remain unaffected by the substrate, while all higher-order modes have disappeared. To reveal the higher-order resonance peaks, we simply multiply the lattice thickness by a factor of $n_{Si}=3.44$. Simulating the structure with the following parameters: period a=3μm and thickness $d^{{}^{\prime }}=n_{\mathrm{Si}}\cdot d=10.32\;\mu m$, we obtain a response similar to that of the first structure with the parameters: period a=3μm, thickness d=3μm, and $n_{I}=n_{III}=1$. This response is illustrated in Figure 12 within a reduced frequency range R∈]0,0.3].

## 4. Conclusions

In conclusion, this investigation underscores the efficacy of a heterogeneous plate endowed with sub-wavelength characteristics in producing multiple transmission reductions, resulting in distinctive W-shaped profiles. This result is achieved through the proficient interplay between discrete and continuum states, along with plasmonic resonance phenomena. Furthermore, it is noteworthy that the resulting band gaps can be tailored to exhibit adjustable bandwidths of up to 34% through the manipulation of opto-geometric parameters, including the angle of incidence θ, grating period *a*, thickness *d*, air gap filling factor $f_{1}=\frac{w}{a}$, and Si Slits filling factor $f_{2}=\frac{g}{a}$. Additionally, careful attention is devoted to the overall scale of the structure to enhance metal absorption across a broad spectral range spanning from the infrared to the visible and ultraviolet regions. The semi-analytical framework based on (RCWA) not only demonstrates a high degree of agreement with numerical simulations using (FEM) but also introduces a new level of versatility compared to conventional (RCWA), enabling the modeling of multilayer gratings featuring an array of apertures. Finally, this envisioned structure holds immense promise for various applications, including selective filters, optical sensors, and ultra-thin perfect absorbers.

## Figures and Tables

**Table 1:** Geometric parameters of the structure.

Parameter	Value (μm)	Description
*a*	3	unit cell period
*d*	*a*	unit cell thickness
$h_{I}$	10a	height of region I (supertrat)
$h_{III}$	10a	height of region III (substrate)
$h_{\mathrm{PML}}$	a/3	PML layer thickness
*w*	a/10	air gap width
*g*	a/5	Si gap width
*b*	a/3	distance between two successive slits

**Table 2:** The positions of the transmission minima at various incidence angles.

$\theta {(}^{\circ })$	*R*
0	$\frac{1}{1+\sin0}=1$
15	$\frac{1}{1+\sin15}\approx 0.794$
30	$\frac{1}{1+\sin30}\approx 0.667$
45	$\frac{1}{1+\sin45}\approx 0.586$

**Table 3:** The position of transmission maxima for a silicon slit array.

*p*	$R_{p}=\frac{p}{2n_{\mathrm{Si}}}$
1	0.139
2	0.278
3	0.416
4	0.556
5	0.694
6	0.833

## Data Availability

The data presented in this study are available on request from the corresponding author.
